# Impact of Freezing on the Microbiological Quality and Physical Characteristics of Buffalo Mozzarella Cheese

**DOI:** 10.3390/ani11123502

**Published:** 2021-12-08

**Authors:** Loredana Biondi, Andrea Fulgione, Federico Capuano, Morena Nappa, Angelo Citro, Donatella Nava

**Affiliations:** 1 Department of Food Inspection, Istituto Zooprofilattico Sperimentale del Mezzogiorno, Portici, 80055 Naples, Italy; loredana.biondi@izsmportici.it (L.B.); andrea.fulgione@izsmportici.it (A.F.); federico.capuano@cert.izsmportici.it (F.C.); morena.nappa@izsmportici.it (M.N.); 2 Reference Center for Traditional Agri-Food Products of the Campania Region (CRIPAT-PAT), 83100 Avellino, Italy; vincenzocitro@inwind.it; 3 Veterinary Services, Local Health Unit of Salerno, Eboli, 84025 Salerno, Italy

**Keywords:** freezing, Buffalo Mozzarella cheese, fresh milk, food safety, microbiological quality

## Abstract

**Simple Summary:**

Buffalo Mozzarella cheese from Campania is made from the fresh milk of the Italian Mediterranean buffalo. In 1996, thanks to its distinctive characteristics (specific environmental conditions and production method), Regulation (EC) No. 1107/96 recognized it as a Protected Designation of Origin product. The limited availability of milk and the increased demand for buffalo mozzarella cheese, especially during the spring-summer period (when milk production is lowest), have induced the use of frozen milk or curd for its production. The aim of this research was to provide preliminary results about the effect of freezing on microbial communities of fresh buffalo milk, curd and Buffalo Mozzarella cheese, and on physical properties (whiteness, hardness, and oxidation state) of Buffalo Mozzarella cheese. The preliminary results obtained have allowed us to conclude that the freezing process if properly carried out, does not compromise the microbiological quality of the products but produces only slight changes of some physical properties.

**Abstract:**

Buffalo Mozzarella cheese from Campania is one of the most worldwide appreciated Italian dairy products. The increased demand for buffalo dairy products and the limited availability of the finest buffalo milk has prompted the diffusion of illicit practices, such as the use of milk, curd, or other products that are frozen or bought at low cost. The aim of this research was to provide preliminary results about the trend of the microbial communities of buffalo milk, curd and Buffalo Mozzarella cheese, during freezing storage of eleven months. At the same time, the alterations of physical properties and the presence of the molecular marker “γ4-casein”, have been investigated. The results showed that freezing reduced the concentrations of the total bacterial count, *Enterobacteriaceae*, coliforms, *Escherichia coli* and yeasts in fresh milk and, the concentrations of the total bacterial count, coliforms, lactic acid bacteria and yeasts in mature curd. In the finished product, no notable decreases were observed, except for lactic acid bacteria. About the γ4-casein, no increase was observed in all matrices. These preliminary results allow us to conclude that the freezing process if properly carried out, does not compromise the microbiological quality and the physical properties of the Buffalo Mozzarella cheese.

## 1. Introduction

“Mozzarella di Bufala Campana”, i.e., Buffalo Mozzarella cheese from Campania (BMC) is one of the worldwide most famous Italian food products.

The term “Buffalo Mozzarella” was regulated by the Italian Presidential Decree of 28 September 1979, which contains the production regulation of BMC and highlights the exclusive use of fresh buffalo milk as the raw material. Later, thanks to the Decree of the President of the Council of Ministers (DPCM) of 10 May 1993, the term “Buffalo Mozzarella” was replaced by “Buffalo Mozzarella of Campania”. Furthermore, in 1996, thanks to its distinctive characteristics (specific environmental conditions and production method), Regulation (EC) No. 1107/96 recognized BMC as a Protected Designation of Origin (PDO) product and, this has been confirmed by the European Community Regulation (Regulation (EC)) No. 103/2008 [1].

According to actual product regulation, BMC is made from fresh milk of the Italian Mediterranean buffalo (*Bubalus bubalis*, Linnaeus 1758) that have to be processed within 60 h of milking and has to be heated at 63 °C (±2 °C) for 15 s. The milk is then allowed to cool to 38–39 °C and the whey starter and rennet are added, thus obtaining the curd. When the curd has ripened (pH 4.5–5.0), it is heated in water at 96 °C (±2 °C) until it becomes shiny and homogeneous, and “strings” of mozzarella are formed. Due to the high temperature reached, this procedure is able to reduce the bacterial load. The strings are then shaped into spheres, which are cooled in cold water and salted [2]. 

Like other dairy products, BMC may be contaminated by several microorganisms which, if present in an amount greater than the allowed limits are able to compromise the safety and the shelf-life of the foodstuffs. Their presence is correlated with the flora of the raw milk, processing conditions and post-heat treatments. These microorganisms can be mainly grouped into pathogenic, spoilage and pro-technological [3,4].

The first group includes *Salmonella* spp., *Listeria monocytogenes*, *Shiga toxin-producing Escherichia coli* (STEC) and coagulase-positive staphylococci enterotoxins. In addition to *Salmonella* spp. and *Listeria monocytogenes*, also *Escherichia coli*, *Coxiella burnetii* and *Brucella* spp. are the most common milk-borne pathogens [5].

The group of spoilage microorganisms comprises total bacterial count, *Enterobacteriaceae*, coliforms, *Escherichia coli*, *Pseudomonas* spp., coagulase-positive staphylococci, psychrotrophic microorganisms, yeasts and molds. All these microorganisms are used as indicators of the hygiene conditions of industrial food processing, and the most important parameters are total bacterial count and *Escherichia coli* quantification. The former indicates the presence of mesophilic aerobic microorganisms of animal origin, and it is associated with microbial quality and freshness of several foods [6]. The latter is a widespread commensal microorganism and its detection could signal the presence of other pathogenic bacteria [7].

The last group includes lactic acid bacteria and yeasts. Unlike the above bacteria, lactic acid bacteria are part of the indigenous microflora of raw milk. They are also used as starter cultures during the fermentation phase, thus, contributing to the final characteristics of fermented cheese products, such as body and texture [8]. Regarding yeasts, although they can be regarded as spoilage microorganisms, they are mainly able to affect the concentrations of volatile aroma compounds in cheese, thus influencing its flavor during ripening [3,4,9].

In addition to microbiological quality, another problem affecting BMC is the annual production of milk which depends on the reproductive activity of the buffaloes. In particular, buffalo is a seasonal polyestrous species which have a high production of milk during the winter and less production during the summer, when the demand is higher. In this way, during the summer, there is an increase in the price of fresh buffalo milk [10].

In order to satisfy the increased demand for BMC during the spring-summer period, and contrarily to what is reported by the actual product regulation, some BMC producers have started to add frozen buffalo milk or curd to the Italian buffalo milk used in BMC production [11].

Recently, owing to the coronavirus disease (COVID-19)pandemic, an Act of the Ministry of Agricultural, Food and Forestry Policies (dated 19 March 2020) temporarily modified this regulation, allowing the use of frozen fresh buffalo milk [12]. 

On the above considerations, the principal goal of this research was to evaluate the microbiological quality (i.e., microbial load of different microorganisms) of frozen fresh buffalo milk and frozen BMC, during 11-month storage under freezing. An SDS PAGE analysis was also carried out in order to verify the presence of a fragment γ4-casein, which has been considered a “molecular marker” of freezing storage by a previous study [13]. The above analyses were also carried out on the products of intermediate processing phases (i.e., heat-treated milk, pre-mature and mature curd). Furthermore, a sensory analysis was carried out on BMC during the entire period of frozen. 

The starting point for this investigation (day 0) was the collection of the fresh matrices which were not subjected to freezing.

The results could provide microbiological and physical evidence on the feasibility of freezing and at the same time could support the “temporary” modification of regulations due to the COVID-19 outbreak. 

## 2. Materials and Methods

### 2.1. Samples and Microbiological Analyses

Sampling was conducted at three dairies located in different Protected Designation of Origin (PDO) reference areas of the Campania region: Caserta, Salerno and Naples. Samples were identified as “X”, “Y” and “Z” according to their different geographic origin. Furthermore, all dairies were chosen for the adequacy of both their premises and equipment. The matrices selected for this study were: raw milk, thermized milk (at 63 °C + 2 °C for 15 s, at all dairies), pre-mature curd (at pH 6.2–6.3), mature curd (at pH 4.5–5.0) and Buffalo Mozzarella cheese from Campania (BMC). 

Twelve aliquots (each of 200 g or 200 mL according to the different matrices, for a total quantity of 2400 g or 2400 mL) of the above matrices were collected from each dairy in sterile bags. One aliquot was analyzed at the time of collection (fresh state: day 0) while the remaining eleven were frozen at −20 °C for eleven months. The storage temperature was monitored by means of the Labguard system (bioMérieux Italia S.p.A; Florence, Italy). Each month, frozen aliquots were thawed at 4 °C for about 24 h [14] and equilibrated at room temperature for 1 h [14], before microbiological analyses. These analyses included the detection of spoilage, pro-technological and pathogenic microorganisms and were carried out according to the official ISO methodologies approved by specific (EC) Regulations and were compared to the limit of detection reported in Table 1. The microorganisms selected for this study were grouped as reported in Table 1.

In addition, aliquots of the matrices were collected from the three dairies at 4-month intervals throughout the study. These results were compared with the microbiological results of the matrices in the “fresh state” collected at the beginning of the study, in order to determine whether the hygiene conditions of the production process were mostly constant.

### 2.2. Detection of Microorganisms

The aliquots were homogenized and subjected to different microbiological analyses, according to specific Reference Methods (Table 1). Briefly, the aliquots were mixed with the appropriate diluents (in a ratio of 1:10), thus obtaining a “stock dilution”. The microbiological analyses of spoilage, pro-technological and pathogenic microorganisms require base-10 scale dilutions in an appropriate solution, in order to enable the microorganisms to be counted. Each analysis was performed in triplicate.

All the counts of the spoilage and pro-technological microorganisms were carried out according to the ISO regulation 7218:2007: “General requirements and guidance for microbiological examinations” [17].

### 2.3. Caseins Extraction and SDS PAGE

The casein extraction and SDS PAGE were performed in order to evaluate the possible increase of the fragment γ4-casein over the entire freezing storage (11 months). The selected matrices were only fresh and frozen buffalo milk, mature curd and BMC because these represent the raw, intermediate and final products of the BMC production process, respectively.

Briefly, each month, frozen aliquots of the above matrices were thawed at 4 °C for about 24 h [14] and equilibrated at room temperature for 1 h [14], before being subjected to the caseins extraction process and SDS PAGE. The caseins extraction from fresh and frozen milk was carried out according to the Aschaffenburg & Drewry method [18]. Curd and BMC were, instead, subjected to the extraction process as reported by EC Regulation 150/2018 [19]. At the beginning of the experiment, also the fresh matrices were subjected to the casein extraction process. After the extraction, all samples were analyzed by SDS PAGE as reported by Alinovi et al. [20]. The protein standard used for molecular weight determination was Precision Plus Protein Standards Dual Color (Bio-Rad, Hercules, CA, USA). A successive densitometric analysis was carried out by using GS-800 (Bio-Rad, Hercules, CA, USA).

### 2.4. Physical Analyses 

The quali-quantitative physical analysis was carried out for each BMC sample, as reported by Alinovi et al. [20]. Seven trained panelists (three men and four women), with previous experiences, regarding the sensory analysis of BMC, were enrolled for this study. The characteristics analyzed were whiteness, hardness, and oxidation state (presence of off-odors). The intensity of the above descriptors was evaluated by the means of a grading system consisting of a numerical scale ranging from 1 (no presence of the attribute) to 5 (strong intensity of attribute). In this way, 35/35 indicates a “strong presence” of the attribute, while 7/35 indicates an “absence” of the attribute. Samples of BMC were portioned in 10 mm cubes for this analysis [20]. The analysis was carried out at the beginning of the research (0 day) and at the end (11 months). Statistical analysis was not performed because the results represent only judgments of the quality of the matrix. 

### 2.5. Other Methods

A grading system based on a numerical scale (3 being excellent and 1 being acceptable) was adopted for the evaluation of the microbiological quality of each dairy with regard to each microorganism (*n* = 10), for the following matrices: fresh milk, mature curd and BMC. Furthermore, in the case of high matrix contamination or pathogen detection, the value of 0 was also added to the grading system. In this way, 30/30 indicates “excellent” microbiological quality of the matrix, while 10/30 indicates “acceptable” quality. The overall microbiological quality of each dairy was evaluated by simply adding up the values obtained for fresh milk, mature curd and BMC (90/90 indicates excellent global microbiological quality).

Statistical analysis was not performed because the above grading systems represent only a judgment about the microbiological analyses of the matrices.

## 3. Results

### 3.1. Microbiological Analyses 

First of all, the data from the microbiological analyses carried out on the aliquots of the different matrices collected at 4-month intervals were comparable to those recorded for the “fresh” matrices collected at the beginning of the research. Thus, there were no significant variations in the hygiene conditions of the production process in any of the dairies (data not shown).

The results of this study showed that no pathogens were detected in any of the matrices over the entire period of freezing storage, while a different situation was observed for spoilage and pro-technological microorganisms.

We focused our attention on fresh buffalo milk, mature curd and BMC because these matrices are the raw, intermediate and final products of the BMC production process, respectively. Data on the microbiological analyses of the other matrices (i.e., thermized milk and pre-mature curd) are reported in Supporting Information (Tables S1 and S2).

**Figure 1:**
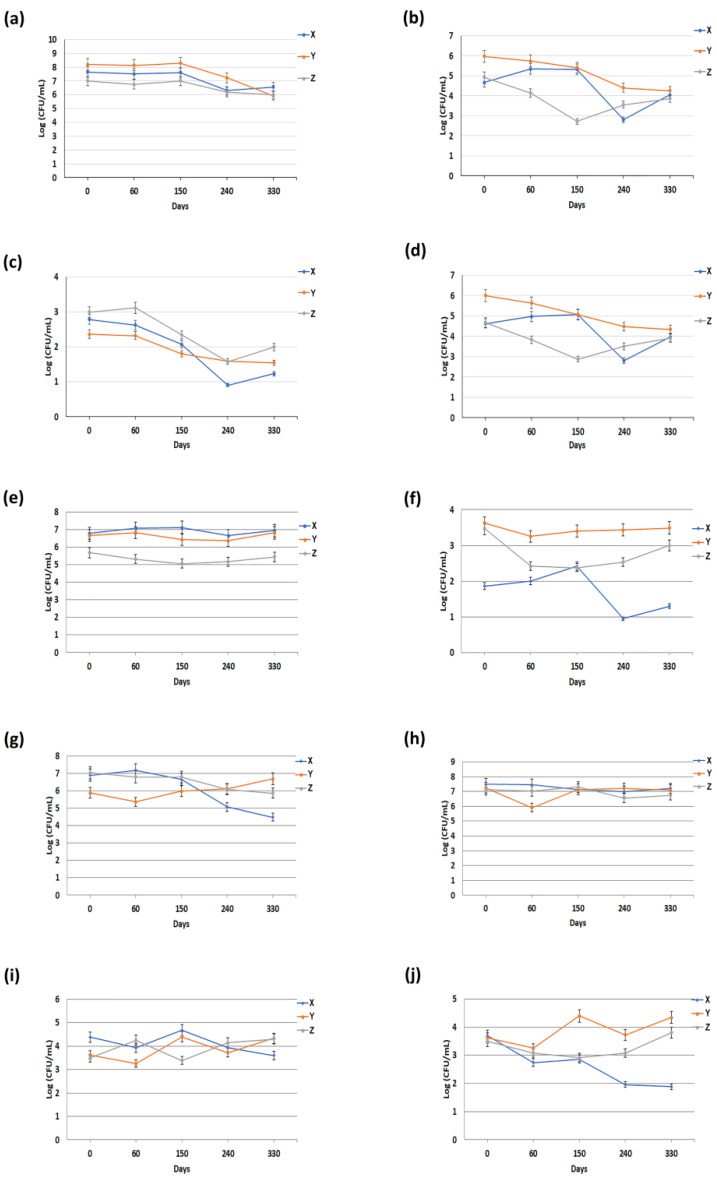
Microbiological data on fresh buffalo milk collected from three different dairies (X, Y and Z). Quantification of: (**a**) total bacterial count; (**b**) coliforms; (**c**) *Escherichia coli*; (**d**) *Enterobacteriaceae*; (**e**) lactic acid bacteria; (**f**) coagulase-positive staphylococci; (**g**) *Pseudomonas* spp.; (**h**) psychrotrophic; (**i**) yeasts and (**j**) moulds. Each datum represents the mean ± SD of the different microbiological analyses, each performed in triplicate.

Figure 1 showed the effect of freezing on buffalo fresh milk. In particular, the freezing process was able to significantly reduce (about 2 logs) the concentrations of the following microorganisms: total bacterial count, coliforms, *Escherichia coli* and *Enterobacteriaceae* over the entire time of the study and in all the dairies (Figure 1a–d). However, a different situation emerged with regard to coagulase-positive staphylococci and *Pseudomonas* spp. Specifically, a decrease in the concentrations of these two microorganisms was observed in two dairies (X and Z), while no decrease was observed in the remaining dairy (Figure 1f,g). Concerning Yeasts, a 1 log decrease in concentration was observed in the fresh milk from dairy X, while a slight increase was detected in the fresh milk from the other two dairies (Figure 1i).

A diverse trend was also noted with regard to mold counts: a significant reduction in the concentration of molds at the end of the analyses (330 days) was only detected in fresh milk collected from dairy X (Figure 1j). Regarding psychrotrophic and lactic acid bacteria, no significant variations were observed during the time of the study, in all dairies (Figure 1e–h).

**Figure 2:**
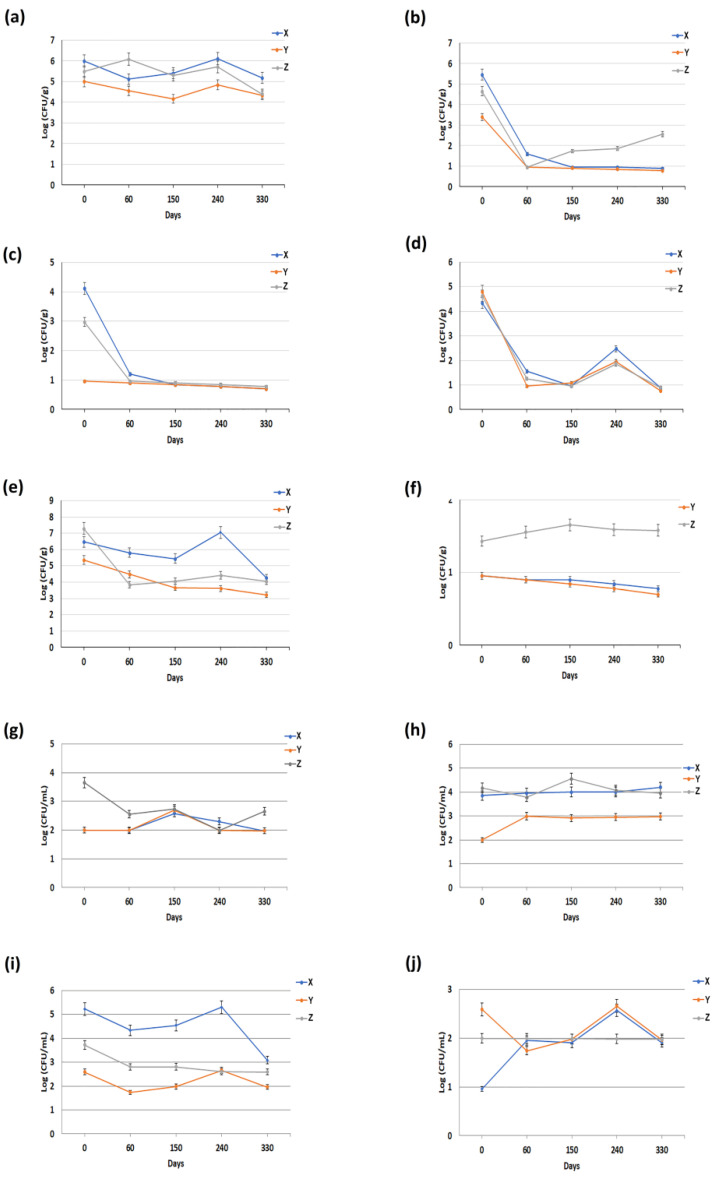
Microbiological data on mature curd collected from three different dairies (X, Y and Z). Quantification of: (**a**) total bacterial count; (**b**) coliforms; (**c**) *Escherichia coli*; (**d**) *Enterobacteriaceae*; (**e**) lactic acid bacteria; (**f**) coagulase-positive staphylococci; (**g**) *Pseudomonas* spp.; (**h**) psychrotrophic; (**i**) yeasts and (**j**) moulds. Each datum represents the mean ± SD of the different microbiological analyses, each performed in triplicate.

The freezing process also had an impact on mature curd (Figure 2). In all dairies, a 1 log reduction was also observed in the total bacterial count (Figure 2a). In addition, a decrease of about 2 logs in the concentrations of coliforms and lactic acid bacteria in the mature curds were observed in all dairies, during the entire period of analysis (Figure 2b,e). Furthermore, the analyses revealed a similar value of coagulase-positive staphylococci concentration in the mature curd from the three dairies (Figure 2f). Comparable concentrations of *Enterobacteriaceae* were observed in all dairies (Figure 2d). With regard to *Escherichia coli*, by contrast, the microbial load was significantly reduced in the mature curd collected from dairies X and Z, while in Y it remained almost constant throughout the study period (Figure 2c). An opposite trend was observed for psychrotrophic microorganisms: the microbial load increased in mature curd collected from dairy Y (about 1 log), while in X and Z it remained constant throughout the research period (Figure 2h). Regarding *Pseudomonas* spp. concentrations, microbiological analysis evidenced comparable results among all dairies, although dairy Z showed about a 1 log difference from the other dairies at time 0 (“fresh state”) (Figure 2g). Freezing reduced the concentration of yeasts by about 1 log in mature curd collected from dairies Y and Z, and by about 2 logs in that collected from dairy X (Figure 2i). Concerning molds, freezing seemed to have no effect on the mature curd collected from dairy Z. However, it reduced the concentration by about 1 log in mature curd collected from dairy Y and increased the concentration by about 1 log in mature curd from dairy X (Figure 2j).

**Figure 3:**
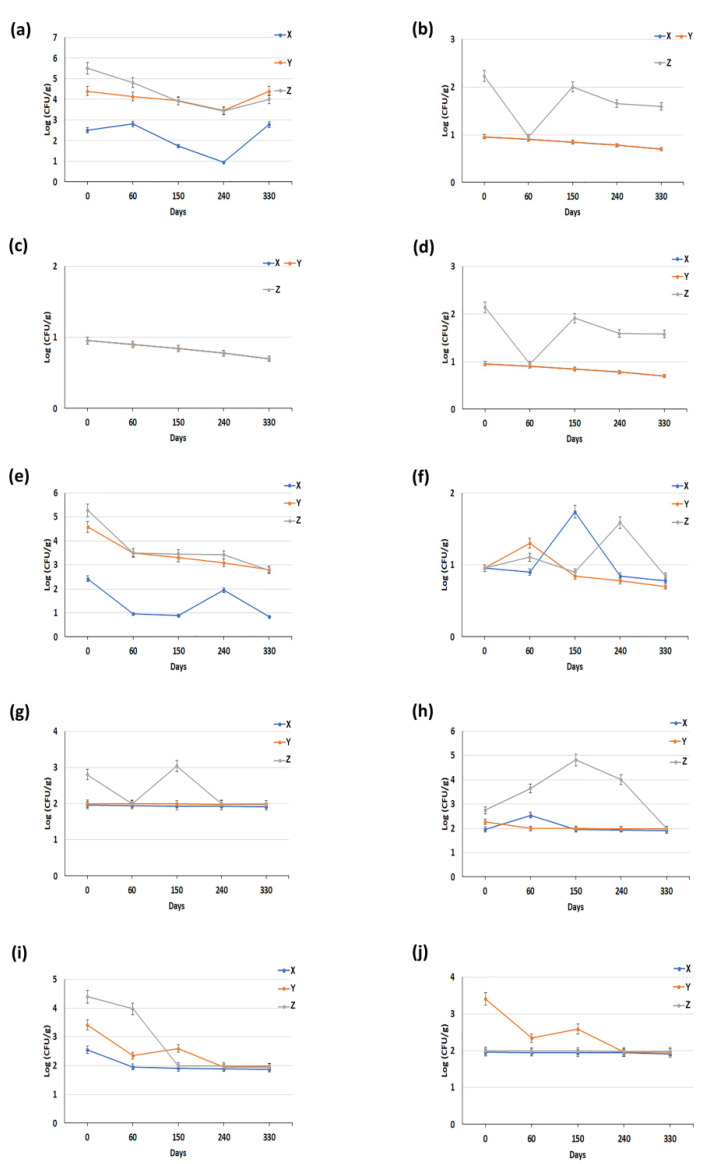
Microbiological data on Buffalo Mozzarella cheese collected from three different dairies (X, Y and Z). Quantification of: (**a**) total bacterial count; (**b**) coliforms; (**c**) *Escherichia coli*; (**d**) *Enterobacteriaceae;* (**e**) lactic acid bacteria; (**f**) coagulase-positive staphylococci; (**g**) *Pseudomonas* spp.; (**h**) psychrotrophic; (**i**) yeasts and (**j**) moulds. Each datum represents the mean ± SD of the different microbiological analyses, each performed in triplicate.

The microbiological analyses of the BMC provided by the three dairies have shown a similar trend about *Escherichia coli*, coagulase-positive staphylococci and psychrotrophic counting (Figure 3c,f,h). A similar and constant trend was also observed for coliforms, *Enterobacteriaceae* and *Pseudomonas* spp. in BMC provided by dairies X and Y, while a reduction of about 1 log was seen in BMC from dairy Z (Figure 3b,d,g). Concerning the total bacterial count, only in the BMC collected from dairy Z was a reduction of about 1 log recorded; in the remaining two dairies, by contrast, the total bacterial counts were constant (Figure 3a). Concerning lactic acid bacteria, a reduction of about 2 logs concentration was observed in all dairies (Figure 3e). By contrast, yeasts were present at different concentrations in the BMC aliquots from the three dairies; however, by the end of the study period (330 days), the higher concentrations had declined to the same level as the lowest (Figure 3i). A similar pattern was also observed in dairies X and Z with regard to mold concentrations (Figure 3j).

Furthermore, the results also revealed that some microorganisms started to multiply in different periods of freeze storage (ranging from 60 to 240 days). For example, in fresh milk, *Escherichia coli*, lactic acid bacteria and *Pseudomonas* spp. showed a peak at 60 days of freeze storage (Figure 1c,e,g), while total bacterial count, yeasts and molds showed a peak at 150 days (Figure 1a,i,j). In mature curd, by contrast, a peak at 240 days was observed for the following microorganisms: total bacterial count, *Enterobacteriaceae*, lactic acid bacteria, yeast and molds (Figure 2a,d,e,i,j), while only *Pseudomonas* spp. showed a peak at 150 days (Figure 2g). Finally, about BMC, it has been observed that there was only a slight decrease in the microbiological parameters, and no significant peaks were detected over the entire study period (Figure 3).

The use of a grading system allowed us to evaluate the microbiological quality of each dairy. The “Overall Microbiological quality” of each dairy was calculated by summing the scores assigned to the matrices selected (fresh milk, mature curd and BMC). Table 2 shows the “Single scores” (values obtained by using the grading system to evaluate the microbiological data on each microorganism in each matrix) and the overall scores. On the basis of our results, we can affirm that all three dairies used fresh milk of good microbiological quality (X: 21/30; Y: 18/30, and Z: 22/30). By contrast, only dairy Y was able to carry out an efficient process of curd maturation, thus obtaining mature curd of “excellent” microbiological quality (28/30). Regarding BMC, dairies X and Y produced an almost “excellent” BMC, reaching values of 29/30 and 26/30, respectively.

The Overall Microbiological quality, obtained by adding the values of the matrices collected from each dairy, indicated that dairies Y (72/90) and X (68/90) performed better than dairy Z (56/90). However, none of the dairies selected achieved the maximum score (90/90), thus evidencing that none of them was able to respect all the hygiene and technological standards required by the BMC production process.

It is important to point out that this study was performed in order to observe and evaluate the microbiological communities present in different matrices collected from three different dairies, and not to select the best dairy or product. Consequently, and also owing to the presence of several confounding factors (geographic location of dairies, geographic origin of the milk, tonnage and month of lactation of buffaloes, environmental factors, etc.), statistical analyses comparing dairies were not carried out.

### 3.2. SDS PAGE

**Figure 4:**
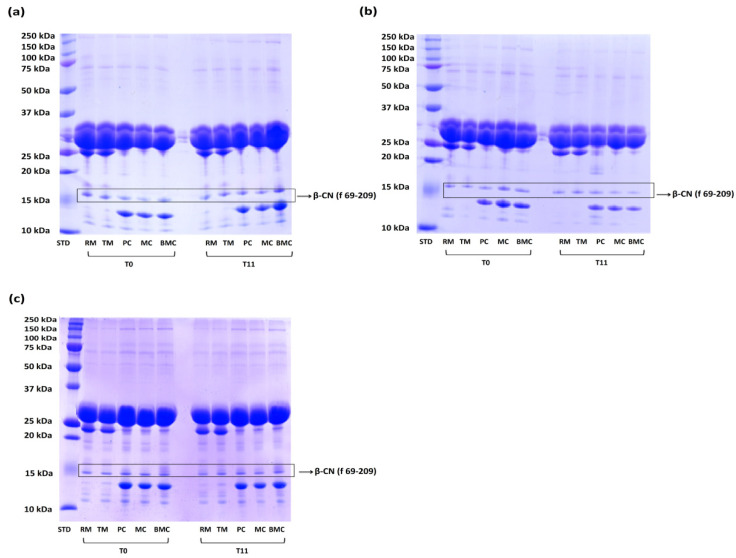
SDS PAGE of the matrices of the three dairies: (**a**) Dairy X; (**b**) Dairy Y; (**c**) Dairy Z. The results reported are referred to the beginning of the experiment (T0; fresh matrices, 0 day) and to the end of the freezing storage (T11; 11 months). The fragment γ4-casein selected for this study has been indicated as “β-CN (69-209)”. The matrices were: RM (raw milk), TM (thermized milk), PC (pre-mature curd), MC (mature curd) and BMC (Buffalo Mozzarella cheese). STD (Standard) is the Protein Molecular Weight Marker (10-250 kDa).

The SDS PAGE allowed us to evaluate the presence of the fragment γ4-casein defined—also defined “B-CN (69-209)”—in fresh and frozen matrices [13]. The presence of this fragment is due to the enzymatic activity of bovine plasmin on β-casein, contained in milk. In a previous study, it has been identified as a “molecular marker” of freezing storage [13]. This analysis showed the presence of the fragment (Figure 4) while the densitometric analyses did not confirm its increase over the freezing storage (Table 3). In Figure 4, it has been reported only the SDS PAGE was carried out at the beginning of the research (T0; 0 day, fresh matrices) and at the end of the freezing storage (T11; 11 months). It is important to highlight this constant trend of the fragment γ4-casein was observed for each month and for all diaries for the entire time of investigation. These data were also supported by a previous study which has performed a SDS PAGE of mozzarella cheese samples until four months of freezing storage [14].

### 3.3. Physical Analysis

The use of a grading system allowed us to also evaluate the physical quality of the BMC produced by each dairy. Table 4 shows the “Single scores” (values formulated by each panelist) about the three different properties selected for this analysis.

The results, obtained by the collection of the judgments, showed that at the beginning of the experiment (0 day), all the three BMC have shown the same physical properties. This is probably associated with the freshness of the products. The single scores collected at the end of the experiment (11 months) have highlighted the changes that occurred during the freezing period. In particular, the whiteness of the BMC moved toward a yellow color thus confirming an increased state of oxidation and, also the hardness increased in particular, the judgments about the physical properties of BMC produced by dairy “Y” were slightly different from the other two dairies. This reflects the same trend of the Overall Microbiological quality results, which have shown that the dairy Y was able to produce an almost “excellent” BMC (Table 2).

## 4. Discussion

Several characteristics, such as the quality and safety of BMC and other fresh cheeses, are associated with microbiological parameters. The trends in the microorganisms selected for this study highlighted two important elements.

First, the different concentrations of the same microorganism in the various matrices—at the time of collection (fresh state: day 0)—shows that the BMC production process was not properly carried out by the dairies, and also that this process was carried out differently by each dairy. In our opinion, the above differences could be explained by the imperfect execution of heat treatment, inefficient cleaning/sanitization, or inadequate process temperatures. The above considerations are also supported by analysis of the “Single scores” and “Overall Microbiological quality”. Specifically, these results indicate that dairies Y and X adhered more strictly than dairy Z to the technological (time, temperature and pH) and hygiene parameters. Moreover, these parameters were more rigorously respected by dairy Y than by dairy X. This conviction emerges from the fact that, although the milk used by dairy Y was of lower hygiene quality than that used by X and Z, the finished product (BMC) of dairy Y was the best. 

Second, the decreased concentration of some microorganisms within the same matrix over the entire period of observation could be linked to the prolonged freezing storage, as reported in several studies [21,22,23,24]. Furthermore, almost all the microorganisms started to multiply in a period ranging from 60 to 240 days of freeze storage (Figure 1, Figure 2 and Figure 3). In accordance with our considerations, this increase could be caused by the freezing process, which can promote the rupture of milk macrophages and neutrophils (thus, releasing the phagocytized bacteria), decompose the bacterial cell aggregates, thus causing the gradual increase of the remaining microorganisms [23,24] Furthermore, about freezing, this destroys only some cell structures. Gram-negative bacteria are usually more vulnerable to freezing than Gram-positive bacteria [25]. Some studies have shown that *Staphylococcus aureus* in milk samples survives freezing [26]. Regardless of these effects, some researchers have reported that thawed products deteriorate more rapidly than the same food stored in a fresh state [27].

In addition, the increase in *Pseudomonas* spp. concentration in all matrices during freeze storage could be associated with the genetic ability of some *Pseudomonas* spp. to synthesize heat-stable proteinases and lipases, both of which are able to attack the protein and lipid components of milk [28].

The SDS PAGE analyses have shown the constant presence of the fragment γ4-casein in all matrices selected, thus not confirming the hypothesis of its variation associated with freezing storage (Figure 4, Table 3). Furthermore, some physical properties (whiteness, hardness and oxidation state) of BMC, were subjected to slight changes (Table 4).

In particular, the panel test allowed us to verify the worsening of both the inner and outer part of the product, thus confirming previous studies [20,29]. About whiteness, its increase toward yellow color during the freezing storage is probable due to the modifications of the milk characteristics; while the increased hardness of the BMC was probably due to casein hydrolysis. The increased oxidation state associated with the development of off-odors, instead, was associated with the oxidation of the lipid phase [30] and also from non-enzymatic browning [20].

## 5. Conclusions

In 1996, the BMC has been recognized as a Protected Designation of Origin (PDO) product by the Regulation (EC) No. 1107/96 and, subsequently, it was confirmed by the European Community Regulation (Regulation (EC)) No. 103/2008 [1]. 

The increased demand for BMC during the spring-summer period (when the production of buffalo milk is less) and the, recent Act of the Ministry of Agricultural, Food and Forestry Policies (dated 19 March 2020) due to coronavirus disease (COVID-19), have induced some BMC producers to use frozen buffalo milk for BMC production [11,12]

In this way, the aim of this research was to investigate the microbiological quality of frozen fresh buffalo milk and frozen BMC, during 11-month storage under freezing and also the presence of the fragment γ4-casein [13].

The results obtained have allowed us to conclude that the freezing process if properly carried out, does not compromise the microbiological quality of the milk, curd and BMC. In particular, our data indicate the feasibility of the freezing treatment and that the mentioned regulatory modification due to the COVID-19 outbreak, did not imply any health risk. 

However, it is important to state that, if dairies want to utilize frozen milk or curd and guarantee the microbiological quality of their product, they should very strictly respect the highest standards of hygiene.

This study needs to be supported by additional research. First of all, further studies need to be carried out in order to evaluate additional parameters of fresh milk, curd and BMC, such as chemical composition, rheological properties, etc. Secondly, species-specific PCR should be performed in order to identify the yeast species, since we only carried out a quantitative microbiological evaluation.

## Figures and Tables

**Table 1:** Reference methods and limit of detection for the microbiological analyses of spoilage, pro-technological and pathogenic microorganisms.

**Spoilage Microorganisms**	**Reference Methods**	**Microbiological Criteria (Limit of Detection)**
Total bacterial count	UNI ^1^ EN ^2^/ISO ^3^ 4833-1:2013	Regulation (EC) No. 853/2004 [4]
Coliforms	UNI ISO 4832:2006	Linee guida per l’analisi del rischio nel campo della microbiologia degli alimenti [15].
*Enterobacteriaceae*	UNI EN/ISO 21528-2:2017	Commission Regulation (EC) No. 2073/2005 [3]
*Escherichia coli* β-glucuronidase positive	UNI ISO 16649-2:2001	Commission Regulation (EC) No. 2073/2005 [3]
Psychrotrophic	UNI ISO 17410: 2019	Conferenza Stato-Regioni 212/2016 [16]; Linee guida per l’analisi del rischio nel campo della microbiologia degli alimenti [15].
*Pseudomonas* spp.	UNI ISO/TS ^4^ 11059:2009 and IDF/RM ^5^ 225:2009	Linee guida per l’analisi del rischio nel campo della microbiologia degli alimenti [15]
Coagulase-positive staphylococci	UNI EN/ISO 6888-2:2004	Commission Regulation (EC) No. 2073/2005 [3]
Yeasts ^+^ and moulds	UNI ISO 21527-1:2008	Conferenza Stato-Regioni 212/2016 [16]
**Pro-Technological Microorganisms**	**Reference Methods**	
Yeasts ^+^	UNI ISO 21527-1:2008	Conferenza Stato-Regioni 212/2016 [16]
Lactic acid bacteria	UNI ISO 15214:1998	Linee guida per l’analisi del rischio nel campo della microbiologia degli alimenti [15]
**Pathogen Microorganisms**	**Reference Methods**	
*Salmonella* spp.	UNI EN/ISO 6579-1: 2017	Commission Regulation (EC) No. 2073/2005 [3]
*Listeria monocytogenes*	UNI EN/ISO 11290-1:2017	Commission Regulation (EC) No. 2073/2005 [3]
*Escherichia coli* O157:H7	UNI EN/ISO 16654:2001	Conferenza Stato-Regioni 212/2016 [16]
Coagulase-positive staphylococci enterotoxins	AOAC ^6^ Official method 2007.06 VIDAS set 2 for detection of staphylococcal enterotoxins.	Commission Regulation (EC) No. 2073/2005 [3]

^1^ (UNI) Italian National Unification; ^2^ (EN) European Norms; ^3^ (ISO) International Standards Organization; ^4^ (ISO/TS) ISO Technical Specification; ^5^ (IDF/RM) International Dairy Federation/Report of Meeting; ^6^ (AOAC) Association of Official Analytical Chemists; (^+^) As reported above, Yeasts can be considered both spoilage and pro-technological microorganisms.

**Table 2:** “Single Scores” and “Overall Microbiological quality” of selected matrices for each dairy.

Matrices	Dairy X	Dairy Y	Dairy Z
Fresh milk	21/30	18/30	22/30
Mature curd	18/30	28/30	18/30
BMC ^1^	29/30	26/30	16/30
Overall Microbiological quality	68/90	72/90	56/90

^1^ (BMC) Buffalo Mozzarella cheese from Campania.

**Table 3:** Densitometric analysis of the matrices of the three dairies: (a) Dairy X; (b) Dairy Y; (c) Dairy Z. The results reported are referred to the beginning of the experiment (T0; 0 day) and to the end of the freezing storage (T11; 11 months). Values are expressed as % of the amount of γ4-casein (β-CN (69-209)) normalized against to αs1-αs2-β CN value.

**Start Time (0 Day)**			
**Matrices**	**Dairy X**	**Dairy Y**	**Dairy Z**
RM (raw milk)	11.783	10.14	11.032
TM (thermized milk)	11.810	10.25	11.012
PC (pre-mature curd)	27.949	40.476	35.62
MC (mature curd)	30.987	50.168	38.021
BMC (Buffalo Mozzarella cheese)	12.059	10.98	11.389
**End Time (T11; 11 Months)**			
**Matrices**	**Dairy X**	**Dairy Y**	**Dairy Z**
RM (raw milk)	12.281	10.245	11.103
TM (thermized milk	12.275	10.17	11.101
PC (pre-mature curd)	28.446	40.486	35.231
MC (mature curd)	31.006	50.698	38.021
BMC (Buffalo Mozzarella cheese)	12.254	10.023	11.658

**Table 4:** “Single Scores” of BMC from each dairy at the beginning (0 day) and at the end of the experiment (330 days).

**Start Time (0 Day)**			
**Parameters**	**Dairy X**	**Dairy Y**	**Dairy Z**
Whiteness	35/35	35/35	35/35
Hardness	8/35	7/35	8/35
Oxidation state	7/35	8/35	7/35
**End Time (11 Months)**			
**Parameters**	**Dairy X**	**Dairy Y**	**Dairy Z**
Whiteness	8/35	12/35	7/35
Hardness	33/35	29/35	35/35
Oxidation state	30/35	27/35	34/35

## Data Availability

The data presented in this study are available on request from corresponding author.

## Supplementary Materials

The following are available online at https://www.mdpi.com/article/10.3390/ani11123502/s1, Table S1: Microbiological data of thermized buffalo milk collected from different dairies. Each data represents the mean ± SD of the different microbiological analyses, each performed in triplicate; Table S2: Microbiological data of pre-mature curd collected from different dairies. Each data represents the mean ± SD of the different microbiological analyses, each performed in triplicate.
