# Supplementary material for: Impact of Freezing on the Microbiological Quality and Physical Characteristics of Buffalo Mozzarella Cheese
Source: Animals (Basel). 2021 Dec 8;11(12):3502. doi: 10.3390/ani11123502 (PMC8698174; doi:10.3390/ani11123502)
Supplement: Supplementary file 1 [file animals-11-03502-s001.zip › animals-1463115-supplementary.pdf]

SUPPLEMENTARY FILES

Impact of freezing on the microbiological quality and physical characteristics of buffalo mozzarella cheese.

Loredana Biondi[1,+], Andrea Fulgione[1,+], Federico Capuano[1-3], Morena Nappa[1], Angelo Citro[2-3], Donatella Nava[1,*].

[1]Department of Food Inspection, Istituto Zooprofilattico Sperimentale del Mezzogiorno, 80055 Portici, Naples, Italy; loredana.biondi@izsmportici.it; andrea.fulgione@izsmportici.it; federico.capuano@cert.izsmportici.it; morena.nappa@izsmportici.it; donatella.nava@cert.izsmportici.it)

[2]Veterinary Services, Local Health Unit of Salerno, 84025 Eboli, Salerno, Italy; (vincenzocitro@inwind.it)

[3] Reference center for traditional agri-food products of the Campania Region (CRIPAT-PAT), 83100 Avellino, Italy; federico.capuano@cert.izsmportici.it; vincenzocitro@inwind.it)

[*]Correspondence: donatella.nava@cert.izsmportici.it; Phone: +39 081 7865338

[+]These authors contributed equally to the study.

Table S1. Microbiological data of thermised buffalo milk collected from different dairies. Each data represents the mean ± SD of the different microbiological analyses, each performed in triplicate.

A) Total bacterial count

Time (days)	Dairy X	Dairy Y	Dairy Z
0	6.505 ± 0.0010	7.322 ± 0.0021	6.447 ± 0.0012
60	6.301 ± 0.0020	6.301 ± 0.0020	6.041 ± 0.0019
150	7.431 ± 0.0018	5.255 ± 0.0012	6.792 ± 0.0018
240	6.707 ± 0.0010	5.732 ± 0.0010	5.880 ± 0.0020
330	6.740 ± 0.0017	5.079 ± 0.0021	6.505 ± 0.0011

B) Coliforms

Time (days)	Dairy X	Dairy Y	Dairy Z
0	4.806 ± 0.0010	6.176 ± 0.0011	4.568 ± 0.0008
60	3.041 ± 0.0018	4 ± 0.0020	3.278 ± 0.0011
150	4.397 ± 0.0012	3.462 ± 0.0016	2.724 ± 0.0015
240	2.886 ± 0.0011	3.342 ± 0.0018	2.863 ± 0.0017
330	2.845 ± 0.0010	2.380 ± 0.0022	2.838 ± 0.0010

C) *Escherichia coli*

Time (days)	Dairy X	Dairy Y	Dairy Z
0	3.806 ± 0.0012	2.602 ± 0.0018	2.662 ± 0.0018
60	3.176 ± 0.0013	2.556 ± 0.0013	2.602 ± 0.0017
150	2.845 ± 0.0015	0.954 ± 0.0017	1.579 ± 0.0009
240	1.908 ± 0.0011	1.491 ± 0.0020	1.568 ± 0.0010
330	0.903 ± 0.0017	0.845 ± 0.0014	0.954 ± 0.0015

D) *Enterobacteriaceae*

Time (days)	Dairy X	Dairy Y	Dairy Z
0	4.653 ± 0.0016	6.113 ± 0.0018	4.778 ± 0.0011
60	3.176 ± 0.0009	4.342 ± 0.0017	2.869 ± 0.0002
150	4.255 ± 0.0012	3.176 ± 0.0007	3.041 ± 0.0018
240	2.799 ± 0.0008	3.431 ± 0.0015	3 ± 0.0019
330	3.041 ± 0.0019	2.255 ± 0.0011	2.954 ± 0.0011

E) Lactic acid bacteria

Time (days)	Dairy X	Dairy Y	Dairy Z
0	5.568 ± 0.0010	6.342 ± 0.0015	3.278 ± 0.0009
60	5.146 ± 0.0015	5.863 ± 0.0017	5.518 ± 0.0010
150	5.322 ± 0.0018	4.672 ± 0.0012	6.079 ± 0.0011
240	5.361± 0.0017	5.041 ± 0.0022	5.672 ± 0.0018
330	5.591± 0.0020	4.672 ± 0.0018	5.342 ± 0.0019

F) Coagulase-positive staphylococci

Time (days)	Dairy X	Dairy Y	Dairy Z
0	0.903 ± 0.0015	0.954 ± 0.0008	2.556 ± 0.0015
60	0.698 ± 0.0008	2.204 ± 0.0015	1.863 ± 0.0021
150	2.204 ± 0.0016	1.740 ± 0.0018	1.591 ± 0.0017
240	0.698 ± 0.0009	1.740 ± 0.0019	1.579 ± 0.0004
330	0.477 ± 0.0010	2.913 ± 0.0017	1.568 ± 0.0009

G) *Pseudomonas* spp.

Time (days)	Dairy X	Dairy Y	Dairy Z
0	1.954 ± 0.0015	1.991 ± 0.0020	7.204 ± 0.0015
60	5.342 ± 0.0018	4.113 ± 0.0018	5.880 ± 0.0020
150	5.568 ± 0.0010	4.146 ± 0.0010	6.079 ± 0.0008
240	1.954 ± 0.0016	4.518 ± 0.0009	5.431 ± 0.0010
330	3.633 ± 0.0019	3.819 ± 0.0010	5.602 ± 0.0011

H) Psychrotrophic

Time (days)	Dairy X	Dairy Y	Dairy Z
0	6 ± 0.0018	6.477 ± 0.0012	7.113 ± 0.0022
60	5.982 ± 0.0019	5.204 ± 0.0013	6.079 ± 0.0010
150	6.041 ± 0.0020	4.832 ± 0.0018	6.707 ± 0.0008
240	6.079 ± 0.0010	5.342 ± 0.0016	6.041 ± 0.0016
330	6.146 ± 0.0012	4.851 ± 0.0020	5.732 ± 0.0018

I) Yeasts

Time (days)	Dairy X	Dairy Y	Dairy Z
0	3.278 ± 0.0008	0.954 ± 0.0016	3.255 ± 0.0015
60	3.477 ± 0.0010	2.599± 0.0010	2.799 ± 0.0009
150	3.255± 0.0012	2.598 ± 0.0011	3.041 ± 0.0020
240	2.591 ± 0.0020	3 ± 0.0020	3.204 ± 0.0017
330	2.662 ± 0.0018	2.596 ± 0.0007	3.732 ± 0.0018

J) Moulds

Time (days)	Dairy X	Dairy Y	Dairy Z
0	4.041 ± 0.0018	0.954 ± 0.0017	1.995 ± 0.0011
60	1.986 ± 0.0008	1.995 ± 0.0019	2.592 ± 0.0020
150	2.592 ± 0.0016	1.986 ± 0.0012	2.591 ± 0.0022
240	1.991 ± 0.0019	1.991 ± 0.0020	1.986 ± 0.0013
330	1.982 ± 0.0018	2.596 ± 0.0012	1.977 ± 0.0010

Table S2. Microbiological data of pre-mature curd collected from different dairies. Each data represents the mean ± SD of the different microbiological analyses, each performed in triplicate.

A) Total bacterial count

Time (days)	Dairy X	Dairy Y	Dairy Z
0	6.146 ± 0.0010	5.579 ± 0.0012	8.477 ± 0.0012
60	5.591 ± 0.0020	5.380 ± 0.0018	5.176 ± 0.0011
150	6 ± 0.0020	5.079 ± 0.0010	4.838 ± 0.0009
240	4.880 ± 0.0022	4.662 ± 0.0017	6.602 ± 0.0014
330	5.568 ± 0.0011	4.653 ± 0.0015	6.973 ± 0.0016

B) Coliforms

Time (days)	Dairy X	Dairy Y	Dairy Z
0	4.908 ± 0.0010	4.113 ± 0.0018	5.079 ± 0.0011
60	3.041 ± 0.0018	4.041 ± 0.0019	1.591 ± 0.0022
150	3.301 ± 0.0020	3.462 ± 0.0021	4.146 ± 0.0012
240	0.845 ± 0.0012	2.929 ± 0.0010	3.755 ± 0.0011
330	2 ± 0.0022	3.301 ± 0.0017	2.556 ± 0.0010

C) *Escherichia coli*

Time (days)	Dairy X	Dairy Y	Dairy Z
0	4.204 ± 0.0016	2.892 ± 0.0018	3.146 ± 0.0014
60	0.845 ± 0.0014	3.041 ± 0.0020	0.954 ± 0.0016
150	2.690 ± 0.0020	1.591 ± 0.0019	2.857 ± 0.0013
240	1.913 ± 0.0012	1.740 ± 0.0021	2.380 ± 0.0018
330	0.477 ± 0.0010	0.954 ± 0.0016	0.903 ± 0.0017

D) *Enterobacteriaceae*

Time (days)	Dairy X	Dairy Y	Dairy Z
0	4.826 ± 0.0011	4.880 ± 0.0019	5.079 ± 0.0010
60	2 ± 0.0023	4.146 ± 0.0011	2.041 ± 0.0018
150	2.863 ± 0.0015	3.477 ± 0.0010	4.176 ± 0.0015
240	0.903 ± 0.0018	2.908 ± 0.0002	3.176 ± 0.0014
330	2.204 ± 0.0018	3.301 ± 0.0019	2.740 ± 0.0011

E) Lactic acid bacteria

Time (days)	Dairy X	Dairy Y	Dairy Z
0	6.255 ± 0.0015	6.602 ± 0.0019	7.518 ± 0.0009
60	5.963 ± 0.0018	5.954 ± 0.0016	4.591 ± 0.0019
150	5.732 ± 0.0019	5.204 ± 0.0017	4.812 ± 0.0020
240	5.612 ± 0.0020	4.913 ± 0.0019	5.322 ± 0.0018
330	4.954 ± 0.0016	5.380 ± 0.0021	4.954 ± 0.0017

F) Coagulase-positive staphylococci

Time (days)	Dairy X	Dairy Y	Dairy Z
0	0.845 ± 0.0013	0.903 ± 0.0016	2.204 ± 0.0015
60	0.903 ± 0.0018	1.544 ± 0.0015	1.806 ± 0.0011
150	1.653 ± 0.0019	0.845 ± 0.0011	1.653 ± 0.0016
240	0.778 ± 0.0019	1.556 ± 0.0009	1.591 ± 0.0018
330	0.698 ± 0.0011	0.698 ± 0.0007	1.579 ± 0.0010

G) *Pseudomonas* spp.

Time (days)	Dairy X	Dairy Y	Dairy Z
0	1.977 ± 0.0010	1.991 ± 0.0018	4.041 ± 0.0019
60	1.982 ± 0.0018	2.146 ± 0.0015	3.041 ± 0.0023
150	1.986 ± 0.0011	3.612 ± 0.0020	3.342 ± 0.0020
240	1.991 ± 0.0019	3.556 ± 0.0015	3.939 ± 0.0010
330	2.204 ± 0.0015	3.785 ± 0.0017	3.113 ± 0.0017

H) Psychrotrophic

Time (days)	Dairy X	Dairy Y	Dairy Z
0	5.079 ± 0.0010	5.491 ± 0.0017	4.857 ± 0.0009
60	4.079 ± 0.0010	2.832 ± 0.0019	4.556 ± 0.0010
150	4.230 ± 0.0019	2.599 ± 0.0009	4.361 ± 0.0019
240	3.740 ± 0.0020	4.462 ± 0.0013	5.230 ± 0.0020
330	5.041 ± 0.0022	1.995 ± 0.0008	5.176 ± 0.0016

I) Yeasts

Time (days)	Dairy X	Dairy Y	Dairy Z
0	5.113 ± 0.0016	2.491 ± 0.0020	3.579 ± 0.0010
60	5.146 ± 0.0012	2.544 ± 0.0017	2.799 ± 0.0010
150	5.477 ± 0.0010	2.542 ± 0.0019	2.806 ± 0.0012
240	3.968 ± 0.0009	2.525 ± 0.0012	2.591 ± 0.0022
330	3.949 ± 0.0007	2.505 ± 0.0015	2.653 ± 0.0020

J) Moulds

Time (days)	Dairy X	Dairy Y	Dairy Z
0	3 ± 0.0025	1.556 ± 0.0015	1.995 ± 0.0012
60	1.991 ± 0.0019	1.963 ± 0.0017	1.991 ± 0.0017
150	1.977 ± 0.0015	1.959 ± 0.0012	2.690 ± 0.0021
240	2.597 ± 0.0011	1.954 ± 0.0016	2.681 ± 0.0020
330	1.991 ± 0.0020	1.929 ± 0.0010	1.977 ± 0.0012
